# Supplementary material for: Sensitivity of collective outcomes identifies pivotal components
Source: J R Soc Interface. 2020 Jun 3;17(167):20190873. doi: 10.1098/rsif.2019.0873 (PMC7328396; doi:10.1098/rsif.2019.0873)
Supplement: Supplementary Information [file rsif20190873supp1.pdf]

Appendix to "Sensitivity of collective outcomes identifies pivotal components"

A Median Voter Model (MVM)

In the canonical median voter model [1], we assume that voters are described by single-peaked, unidimensional preference function and that they vote according to them. This has been proposed as a model for voting in political science and for how supply-demand curves determine market prices. The median position is special because it determines the only outcome for which it is possible to obtain a simple majority against all alternatives. Here, we map this idea to a reduced, statistical model.

Our formulation, the MVM, consists of an odd number of N Random (R) voters with a single special voter, labeled Median (M), that is guaranteed to vote in the majority. More generally, we can interpolate between a perfect median and set of N random voters by setting a probability α that M votes in the majority and $1-\alpha$ that M votes randomly. The remaining $N-1$ R's are random. This model presents a simple testing ground for exploring the information geometry of a system with a unique, statistically well-defined median voter that can be range from random ($\alpha = 0$) to perfect median ($\alpha = 1$).

The probability distribution defined by the MVM cannot be exactly captured by a pairwise maximum entropy (maxent) model (see Methods). We recall that pairwise maxent models can be derived by maximizing the entropy of the model $S = -\sum_s p(s)\ln p(s)$ while constraining the single component and pair component distributions, $p(s_\mathrm{i})$ and $p(s_\mathrm{i}, s_\mathrm{j})$, respectively, to match the data. From the maxent perspective, the MVM is equivalent to specifying that M be perfectly correlated with the majority of R's, a correlation of the form,

$$\left\langle s_\mathrm{M} \frac{\sum_{\mathrm{i}=1}^{N-1} s_{\mathrm{R_i}}}{\left|\sum_{\mathrm{i}=1}^{N-1} s_{\mathrm{R_i}}\right|} \right\rangle = 1. \tag{S.1}$$

In general, this nonlinear correlation cannot be written as the linear combination of pairwise correlations. Thus, the MVM serves as an example of a model for which the pairwise maxent model, by definition, could only be an approximation, and it provides a test to see if our approach can capture the essential features of the system.

The MVM can be solved numerically for large N by exploiting the symmetry between the Random voters, allowing for fast enumeration of the entire partition function time linear with system size. This solution will be discussed elsewhere, but in Figure S.1 we show numerical results from such a calculation [2]. We find that the eigenvalue of the Median subspace grows linearly with N whereas the eigenvalue for the Random voters grows much slower. Along with this divergence, we find that the asymmetry monotonically grows for the Median at the exclusion of Random voters (since it is normalized). Thus, the MVM presents a model where the Median voter quickly becomes the exclusively dominant voter for large systems, validation of our minimal approach relying on pairwise maxent models.

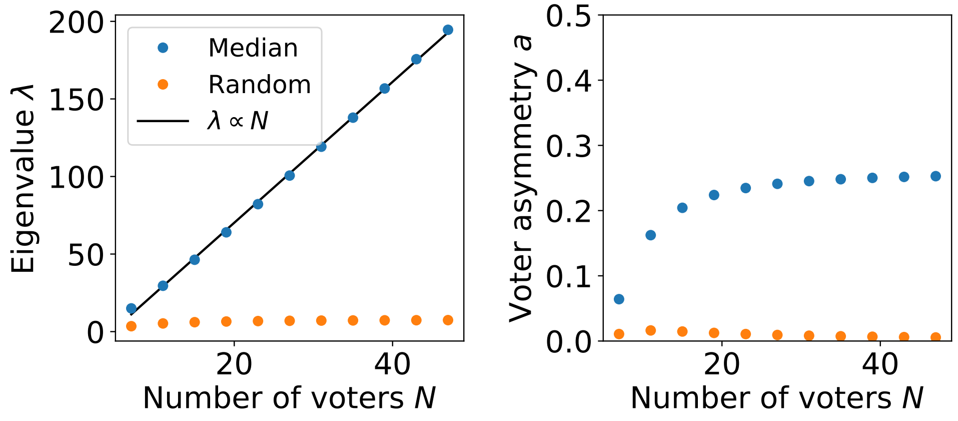

Figure S.1: Voter eigenvalues and asymmetries as a function of system size for the MVM. The subspace eigenvalue for Median grows linearly with system size while its asymmetry asymptotically dominates over Random voters' [2].

B Fitting the pairwise maxent model

We model the probability distribution of votes using a pairwise maxent approach [3]. We begin by maximizing the information entropy $S = -\sum_s p(s) \ln p(s)$ while ensuring that the model match the pairwise correlations calculated over the K data points [4],

$$\langle s_{\rm i} s_{\rm j} \rangle = \langle s_{\rm i} s_{\rm j} \rangle_{\rm data} \tag{S.2}$$

$$\sum_s s_{\rm i} s_{\rm j}\, p(s) = \frac{1}{K} \sum_{\rm k=1}^{K} s_{\rm i}^{\rm k} s_{\rm j}^{\rm k}, \tag{S.3}$$

where the left hand sum is over all possible configurations of the binary vector s, and the right hand sum is over all K observations in the data. Going through the usual calculation [5], we derive the pairwise maxent model

$$p(s) = e^{-E(s)} \big/ Z. \tag{S.4}$$

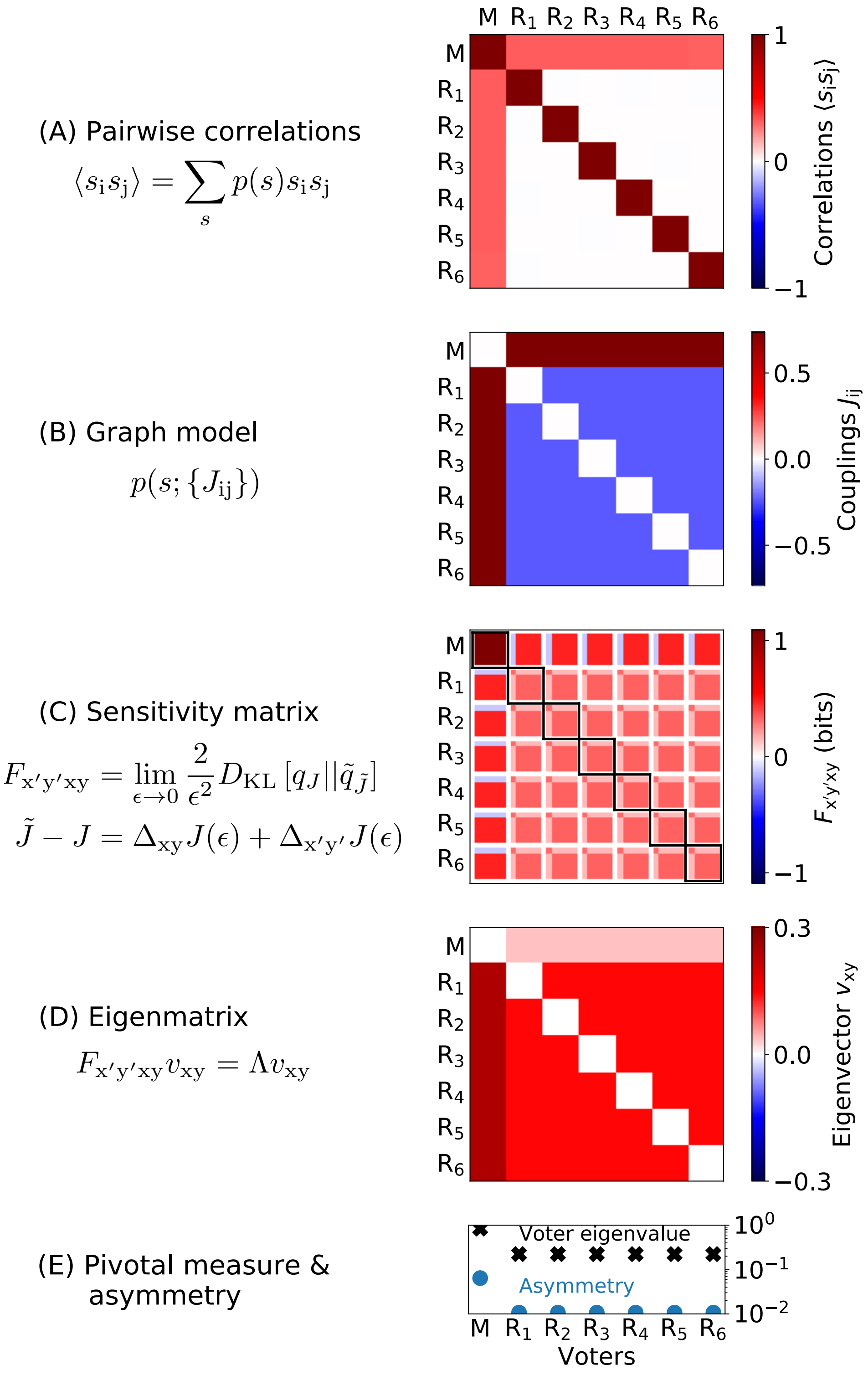
(A) Pairwise correlations
$\langle s_i s_j \rangle = \sum_s p(s) s_i s_j$
M R1 R2 R3 R4 R5 R6
Correlations $\langle s_i s_j \rangle$
1
0
−1
(B) Graph model
$p(s; \{J_{ij}\})$
Couplings J_{ij}
0.5
0.0
−0.5
(C) Sensitivity matrix
$F_{x'y'xy} = \lim_{\epsilon \to 0} \frac{2}{\epsilon^2} D_{KL} [q_J || \tilde{q}_{\tilde{J}}]$
$\tilde{J} - J = \Delta_{xy} J(\epsilon) + \Delta_{x'y'} J(\epsilon)$
$F_{x'y'xy}$ (bits)
1
0
−1
(D) Eigenmatrix
$F_{x'y'xy} v_{xy} = \Lambda v_{xy}$
Eigenvector v_{xy}
0.3
0.0
−0.3
(E) Pivotal measure & asymmetry
Voter eigenvalue
Asymmetry
10^0
10^{-1}
10^{-2}
M R1 R2 R3 R4 R5 R6
Voters

Figure S.2: Overview of method for identifying pivotal voters for the $N = 7$ Median Voter Model to complement Figure 1. (A) Taking the matrix of pairwise correlations, (B) we solve a pairwise maxent model to learn the probability distribution $p(s; \{J_{\mathrm{ij}}\})$ parameterized by the couplings J_{ij}. (C) We calculate the FIM for $q(k)$, the probability of k votes in the majority, measuring the sensitivity of $q(k)$ to changes in voter behavior. The perturbation to the vector of couplings determined by Eqs 1 and 2 are denoted as $\Delta_{\mathrm{xy}} J$. The matrix is segmented into 6x6 blocks for readability. The variation in the entries of the FIM clearly indicates the unique role of the median. Each entry $F_{\mathrm{x'y'xy}}$ of the FIM shows how quickly $q(k)$ changes when two pairs of voters (y becomes x and y′ becomes x′) are changed together. When at least one index is M, we find values different from when only R's are involved. (D) The principal eigenvector of the FIM v_{xy}, reshaped into an "eigenmatrix." (E) The asymmetry a_{y} measures the difference in perturbations localized to a specific voter vs. all its neighbors in turn. The principal subspace eigenvalues, computed from each outlined diagonal block in panel C, give our pivotal measure after normalization.

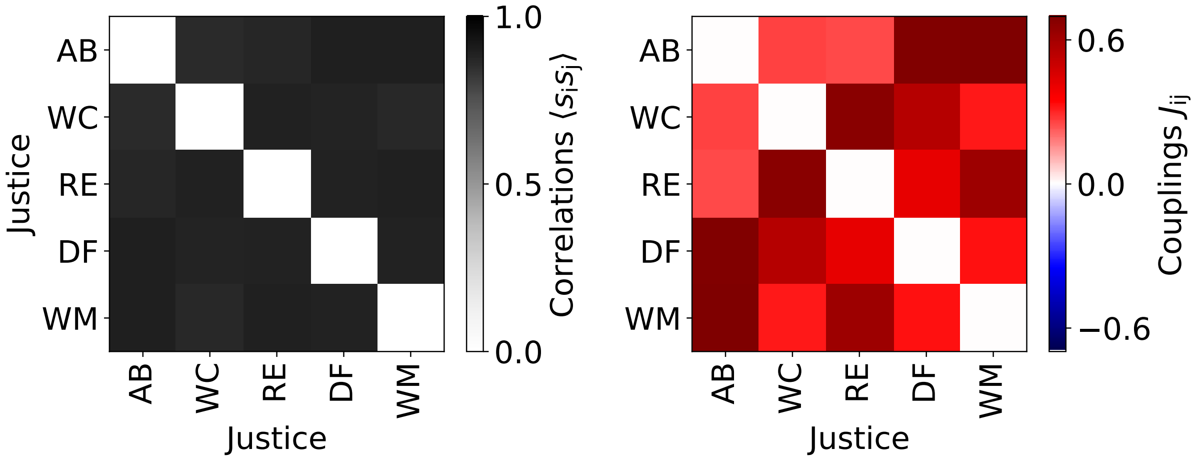

Figure S.3: Pairwise correlations and couplings for AK Supreme Court.

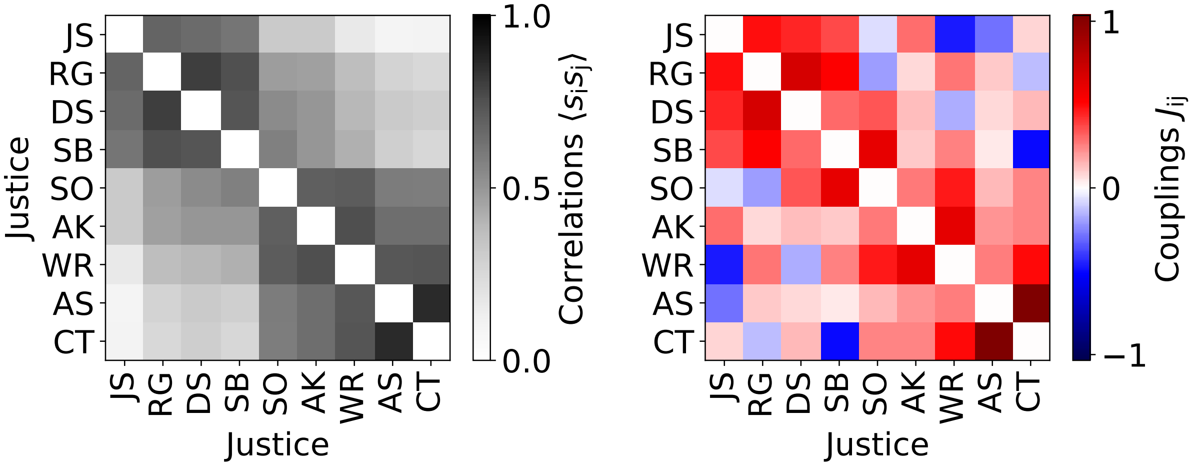

Figure S.4: Pairwise correlations and couplings for US Supreme Court.

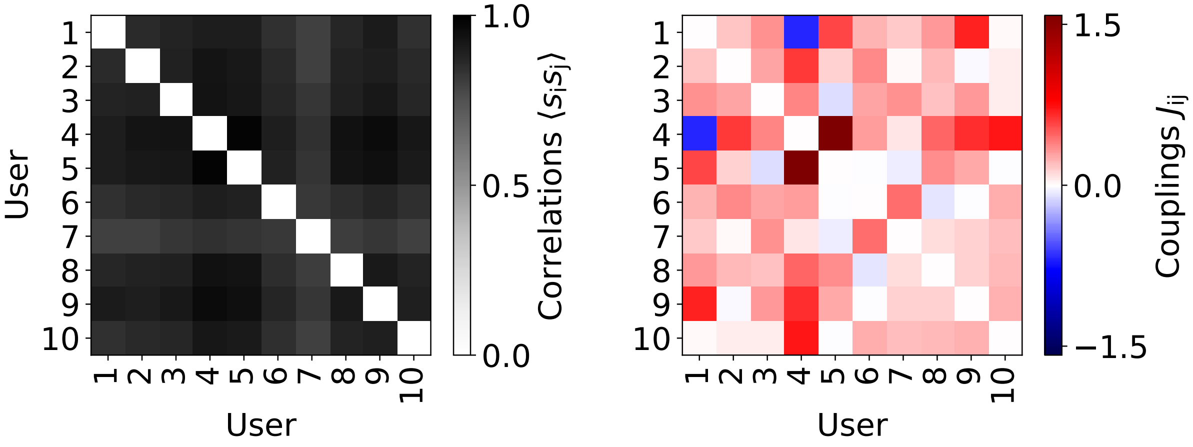

Figure S.5: Pairwise correlations and couplings for Twitter K-pop community.

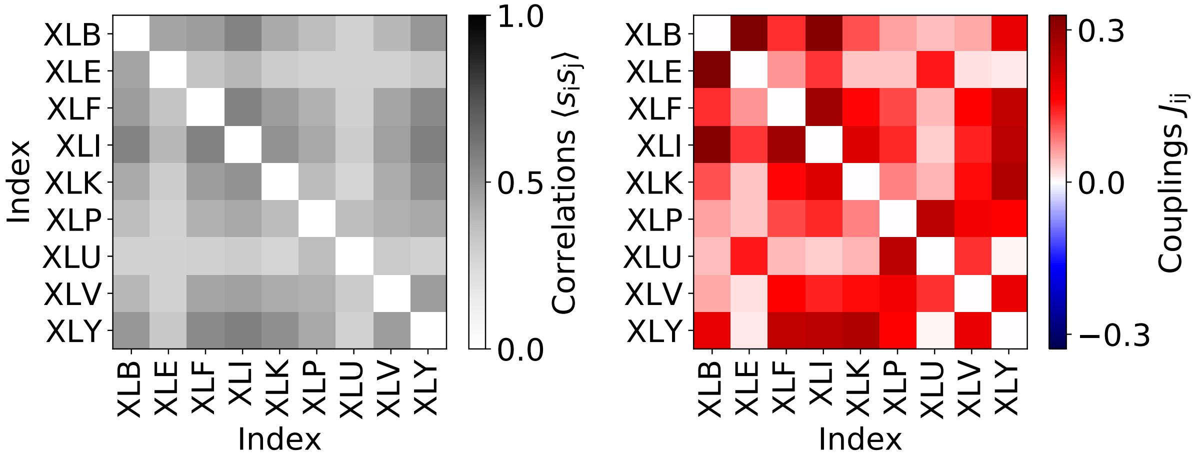

Figure S.6: Pairwise correlations and couplings for S&P SPDR.

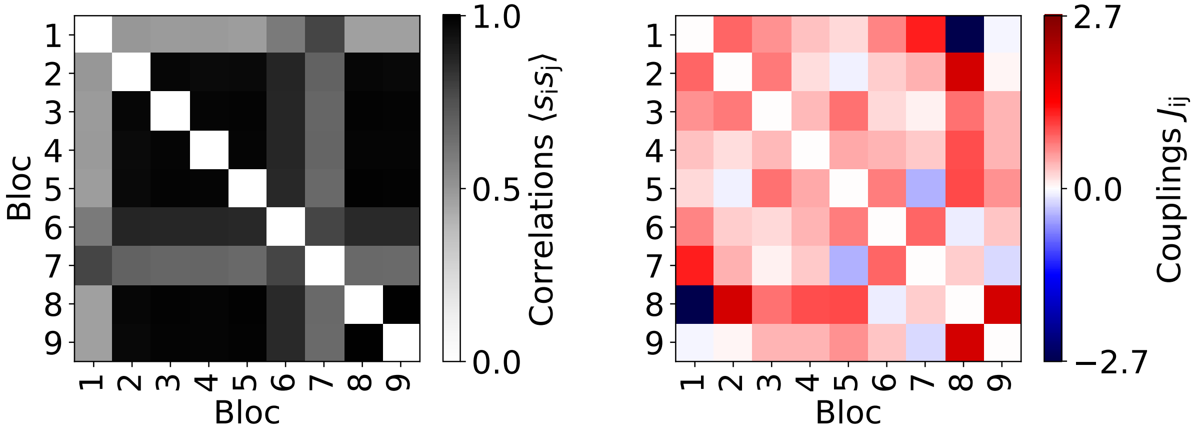

Figure S.7: Pairwise correlations and couplings for CA Assembly 1999 session. Composition of blocs is given in Figure S.14.

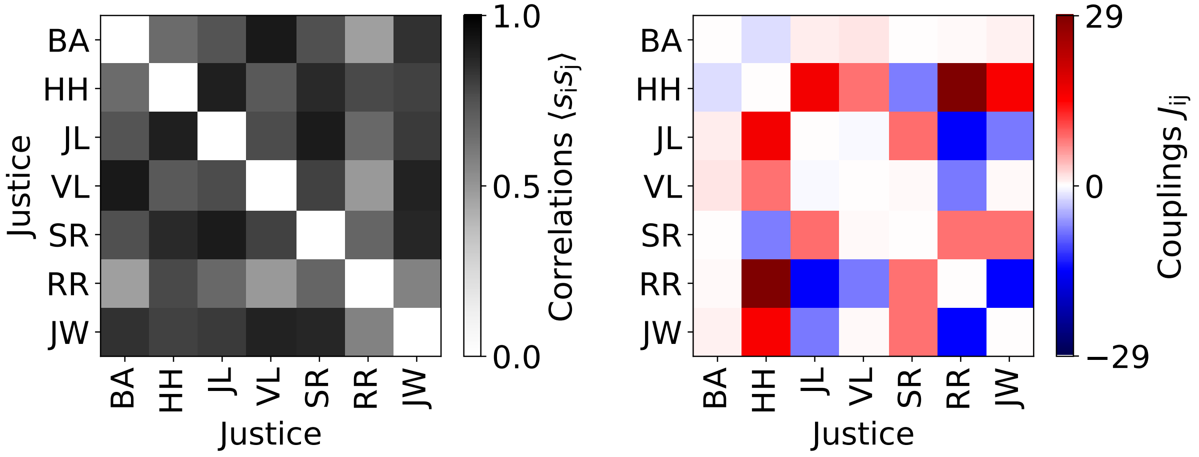

Figure S.8: Pairwise correlations and couplings for NJ Supreme Court. The initials stand for Barry T. Albin, Helen E. Hoens, Jaynee, LaVecchia, Virginia Long, Stuart Rabner, Roberto A. Rivera-Soto, and John E. Wallace Jr.

Eq S.4 is normalized by the "partition function"

$$Z = \sum_{s} e^{-E(s)}, \tag{S.5}$$

and contains the energy functional of the form

$$E(s) = -\frac{1}{2} \sum_{\mathrm{i,j}=1}^{N} J_{\mathrm{ij}} s_{\mathrm{i}} s_{\mathrm{j}}. \tag{S.6}$$

The "couplings" J_{ij} are numerically solved such that the model matches the pairwise correlations.[1] In this sense, the couplings are not fit to the data, but are given exactly by the pairwise correlations in the data. For the small systems that we consider, these couplings can be found exactly by explicit calculation of the pairwise correlations and standard numerical optimization techniques as implemented in the Convenient Interface for Inverse Ising, or ConIII [5]. For the MVM, the problem can be simplified because there are only two types of couplings corresponding to the two types of correlations between Median and Random and between Random voters. All the shown examples from the SI, Figs. S.3–S.8, have least-squares fit norm errors to the

[1]The keen reader may note that we did not constrain the average of each element of the vector $\langle s_{\mathrm{i}} \rangle$, so we did not explicitly fix the individual marginal distributions in this abbreviated derivation. Indeed, we assumed that the averages were 0 because we were only interested in majority-minority dynamics so each vote equally likely to be either up or down, $\langle s_{\mathrm{i}} \rangle = 0$. As a result, the "fields" h_{i} in the complete energy function $E(s) = -\frac{1}{2} \sum J_{\mathrm{ij}} s_{\mathrm{i}} s_{\mathrm{j}} - \sum h_{\mathrm{i}} s_{\mathrm{i}}$ are 0 as is assumed in Eq S.6. In other words, the maxent distribution when fixing only the pairwise correlations shows symmetry about the two possible orientations -1 and 1.

pairwise correlations of $< 10^{-9}$. Thus, the relatively small system size ensures that straightforward enumeration techniques can be used to solve directly for the pairwise maxent models that correspond to the data.

Across all the system that we study, we find that the pairwise maxent model captures well higher-order features of the data even when only fit to the pairwise correlations. To characterize this fit, we use a property of maxent models. The entropy of the maxent model always decreases with the inclusion of additional constraints such that entropy is largest when all N components are treated as independent $S_1 = \log_2 N$ bits and minimized when the entire probability distribution of the data is fit exactly $S_N = S_{\mathrm{data}}$, where S_{i} is the entropy of the model matching all correlations up to and including order i.[2] Thus, as we increase the number of parameters, we impose higher-order structure, and we monotonically approach the entropy of the data. This suggests as a measure for comparing maxent models, the total multi-information captured [3]

$$I = \frac{S_1 - S_2}{S_1 - S_N}, \tag{S.7}$$

which varies from 0 (no improvement beyond the independent model) to 1 (exact fit to the data). For the examples considered in the main text, the pairwise maxent model serves as an excellent fit, capturing over 94% of the multi-information in all cases. The pairwise maxent model captures over 98% of the multi-information for the $N = 7$ MVM. Overall, the pairwise maxent model is a minimal but convincing approximation of the ensemble statistics of the examples we consider [7, 8].

C Specifying the Fisher information (FI) metric

If we have a statistical model described by a probability distribution $p(s;\theta)$ over a set of discrete states s and parameterized by parameters θ, how do we measure how different one model is from another? The Kullback-Leibler (KL) divergence is one such measure that tells us how much information is necessary to reach a distribution $p(s;\tilde{\theta})$ if we know $p(s;\theta)$ [9, 10],

$$D_{\mathrm{KL}}[p(s;\theta)||p(s;\tilde{\theta})] = \sum_s p(s;\theta) \ln\left(\frac{p(s;\theta)}{p(s;\tilde{\theta})}\right). \tag{S.8}$$

In the limit where the two distributions are infinitesimally close to one another, the KL divergence becomes a metric. The constant and linear terms go to zero, and the first nonzero term is the curvature of the divergence, the Hessian, which is also known as the Fisher information (FI),

$$F_{\mathrm{ij}} = \left.\frac{\partial^2 D_{\mathrm{KL}}[p(s;\theta)||p(s;\tilde{\theta})]}{\partial\tilde{\theta}_{\mathrm{i}}\partial\tilde{\theta}_{\mathrm{j}}}\right|_{\tilde{\theta}_{\mathrm{i}}=\theta_{\mathrm{i}},\tilde{\theta}_{\mathrm{j}}=\theta_{\mathrm{j}}}. \tag{S.9}$$

[2] For how to estimate the entropy of the data see references [3] or [6]. Calculating an unbiased estimate of the entropy of the data can be an issue for sparse samples, but is straightforward for the relatively large number of samples we have given the small systems.

Thus, the FI is a description of how quickly the probability distribution changes if we move along various directions in parameter space. Because it is a metric, the eigenvectors of the Hessian correspond to orthogonal directions in the tangent space of the model manifold, where the eigenvalues describe how quickly the manifold is varying along these directions.

The FI also measures how much information about a parameter is in a random sample [9]. When a parameter is extremely sensitive to the distribution of data, then the information shared is high, whereas when it is fairly insensitive the information shared is low. This is described formally by the Cramér-Rao bound, which sets a lower bound on the precision of an unbiased estimator for the parameters [9]. This picture, more formally, has been used as technique for model reduction, removing degrees of freedom in parameter space to which the system is insensitive [11]. Here, we focus on the sensitive degrees of freedom, using them to identify components interesting because their behavior is precisely determined by the statistics of the data, or equivalently on whose behavior collective statistics depend the most sensitively.

We propose using as parameters aspects of the system that provide transparent insight into how perturbation of the parameters affects the system. In physical systems, it is natural to consider the couplings J_{ij}, or more generally the Lagrangian multipliers from the maximum entropy formulation, as the parameters by which to control the system because they are experimentally accessible (e.g., a magnetic system can be tuned by an applied field or by changing temperature that modulates all couplings by a factor). For a statistical model of a social system, however, the meaning of the terms in the energy functional are opaque and often nontrivial. In other words, we do not know how to access the coupling parameter J_{ij}. Certainly, we could be methodical about it, calculate the corresponding changes in the set of pairwise correlations for a perturbation in J_{ij}, but that would result in changes across all pairwise correlations in varying amounts. This change may be difficult to effect in a social system when opportunities for control are often limited.

This impracticality suggests a different approach, where we instead consider how the measured behavior of the system might be perturbed directly since these are straightforward to measure. This reasoning leads us to consider as parameters the observables. Formally, the observables for a maxent model are the conjugate variables to the Langrangian multipliers as given by the Legendre transform [12]. There is no difference in knowing one or the other: the transformation is a one-to-one mapping. For the pairwise maxent model, the "natural parameters" are the couplings and their conjugate the pairwise correlations

$$J_{\mathrm{ij}} \Leftrightarrow \langle s_{\mathrm{i}} s_{\mathrm{j}} \rangle \tag{S.10}$$

$$-\ln Z(\{J_{\mathrm{ij}}\}) + S(\{\langle s_{\mathrm{i}} s_{\mathrm{j}} \rangle\}) = \sum_{\mathrm{i}=1}^{N-1} \sum_{\mathrm{j}>\mathrm{i}}^{N} J_{\mathrm{ij}} \langle s_{\mathrm{i}} s_{\mathrm{j}} \rangle \,. \tag{S.11}$$

Eq S.11 states the well-known relation that the "free energy," $-\ln Z$, is the Legendre transform of the Shannon entropy, having set the units from Boltzmann's constant and temperature $k_{\mathrm{B}}T = 1$. By working in the space of observables, we do not lose any information — indeed the model started with the observables in the first place — but find a more amenable representation.

In the main text, we take one further step by choosing to consider changes to the observables that are interesting as specified in Eq 1. For example, it is a common thought experiment in discussing Supreme Court voting to imagine how the system would change if the justices were different. The justices could be different in any which way, but we narrow the range of possible perturbations substantially by focusing on relative voting records, restricting ourselves to the range of behavior already observed in a system. It makes intuitive sense to ask how the Court would change if Justice Scalia were to vote more like Justice Thomas because their behaviors are specified by the voting record, but it requires much more work to determine what would happen if Scalia were to vote more like a judge picked from the appellate courts. There is no reason such a counterfactual could not be entertained in principle, but it would require modeling that judge's votes on the same set of cases that Scalia voted on. We restrict ourselves from considering such open-ended questions, leaving them as potential extensions of our work. Importantly, we choose perturbations that are localized to particular components, interpretable in their mapping to behavioral changes, and applicable across a wide range of systems.

Another advantage of treating observables as parameters is that it offers some independence from the choice of model. As a simple example of the distinction between treating an observable as the parameter or a term in the energy function, consider the biased coin. With probability p the coin flips heads and with probability $1-p$ it flips tails. If the parameter is the bare observable, the average coin flip, that is equivalent to changing p up to a constant factor.

$$\tilde{p} = p + \epsilon \tag{S.12}$$

Taking the transformation in Eq S.12, we calculate the FI to find (Figure S.9)

$$F = \frac{1}{\log 2}\left[\frac{1}{p} + \frac{1}{1-p}\right]. \tag{S.13}$$

The FI diverges at the boundaries of the parameter space $p=0$ and $p=1$ because that is where a finite change in p can lead to a diverging information distance. Closer to the perturbation considered in the main text, we could insist that the coin "mimic" a perfectly biased coin such that

$$\tilde{p} = \epsilon + (1-\epsilon)p \tag{S.14}$$

Under this scenario, the Jacobian captures the fact that the coin's bias makes no finite jump near $p=1$, but changes ever more slowly as it approaches a perfectly biased coin. As a result,

$$F = \frac{1}{\ln 2}\left[\frac{1}{p} - 1\right], \tag{S.15}$$

which goes to zero at $p=1$.

Now, consider a maxent version of this problem which is the nonlinear transformation

$$p = [\tanh(x) + 1]/2 \tag{S.16}$$

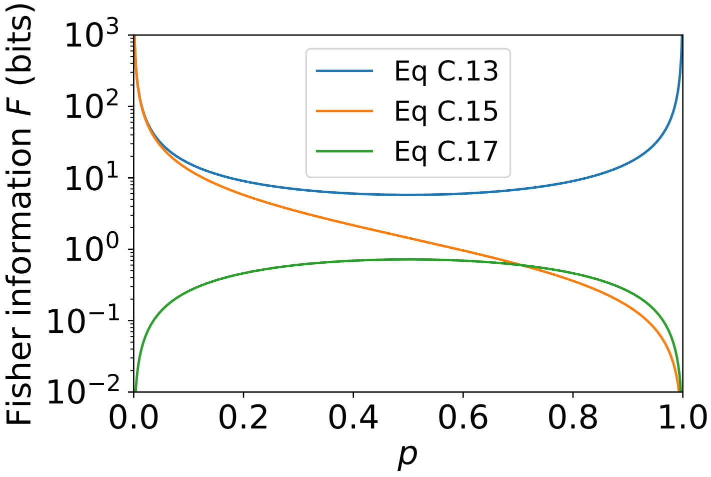

Figure S.9: Fisher information for biased coin according to different choices of perturbation: changing p directly (Eq S.13), substitution with a biased coin (Eq S.15), and changing the "field" (Eq S.17).

where the field determining the bias is x and

$$F = \frac{\operatorname{sech}(x^2)}{2 \ln 2}. \tag{S.17}$$

In contrast with using p as the parameter, the quantity in Eq S.17 peaks at $p = 1/2$ and decays to 0 at the boundaries, and an infinite change in x is necessary to reach $p = 0$ and $p = 1$. Of course, we could have chosen any possible model, choosing instead of the maxent transformation in Eq S.16, our favorite nonlinear transformation. Thus, by choosing our favorite model, we would end up effectively specifying the FI. This is generally not an issue if one cares about measuring the relationship between a particular model and the data, but it does become an issue if one cares more about the statistics of the data rather than of the particular model specified.

If we restrict ourselves to perturbing p, we ensure that the choice of perturbation does not depend on the choice of model. Additionally when the probability distribution is matched exactly, there is no dependence on the model class. In the special case of the biased coin, the probability distribution is specified exactly by a single parameter. Assuming we can measure it with infinite precision and we have a model that can fit the measured p exactly (e.g., a model limited to $0 \leq p \leq 1/2$ does not count), the calculation of FI — whether Eq S.13, S.15, or some other choice — is concretely defined. More generally, data resolution is not perfect and so we must infer probabilities for configurations that we have not observed. As an example, the pairwise maxent approach assumes that the pairwise marginal distributions are known exactly, but the higher-order joint probabilities are assumed to conform to the maxent principle. When any such model feature is used in the calculation of the FI, clearly the FI will depend on the assumptions of the model. For the pairwise maxent model, this means that the calculation of FI

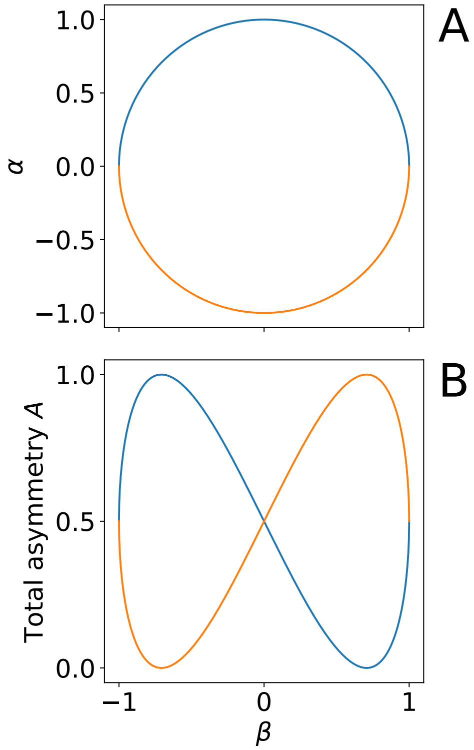

Figure S.10: Total asymmetry for the binary system specified by the 2x2 matrix in Eq S.19.

on features of the distribution that are constructed explicitly from pairwise marginals matches the data exactly, but in general the distribution of majority-minority divisions does depend on the maxent assumptions. This is not always unfavorable: if the higher-order terms decrease in importance such that they behave as small perturbations on top of the pairwise model, the FI will show weaker dependence on these corrections [8]. Thus, we propose a generalizable approach that links minimal, maxent models with a simple class of perturbations defined on the range of relative component behavior.

D Measure of asymmetry

As a way of measuring the heterogeneity between the components of a system, we calculate the asymmetry of the "eigenmatrices" of the Fisher information matrix as defined in Figure 1D. Given that the matrices are normalized $\sum_{\mathrm{xy}} v_{\mathrm{xy}}^2 = 1$, the total asymmetry can be written

$$A = \frac{1}{2}\left(1 - \sum_{\mathrm{xy}} v_{\mathrm{xy}} v_{\mathrm{yx}}\right). \tag{S.18}$$

Thus, we might think of the asymmetry as a measure of correlation between the entries in the upper triangle and lower triangle of the eigenmatrix. When they are perfectly correlated such that the matrix is symmetric, $A = 0$. If the entries $v_{\rm xy}$ are completely uncorrelated with their partners in the transpose $v_{\rm yx}$, then $A = 1/2$. When they are anti-correlated, the summation in Eq S.18 can become negative and $A > 1/2$.

As an example, consider the asymmetry for a 2x2 matrix

$$\begin{pmatrix} 0 & \alpha \\ \beta & 0 \end{pmatrix} \tag{S.19}$$

The normalization constrains α and β to the unit circle,

$$\alpha = \pm\sqrt{1-\beta^2}. \tag{S.20}$$

In Figure S.10A, we have colored the upper and lower halves of this circle (for positive and negative values of α) by different colors. Now calculating the total asymmetry,

$$A = \frac{1}{2} \mp \frac{1}{2}\beta\sqrt{1-\beta^2}. \tag{S.21}$$

We plot Eq S.21 in Figure S.10B and again color the curves differently depending on the half of the unit circle that we are tracing out. When $\beta = -1$, normalization asserts that $\alpha = 0$ and the total asymmetry $A = 1/2$. As we increase β, we can follow α along the positive (negative) route which leads to maximization (minimization) of A at $\beta = -1/\sqrt{2}$. As we keep increasing to $\beta = 0$, we return to $A = 1/2$ and have effectively swapped the roles of $\alpha \to -\beta$ and $\beta \to \alpha$ (a rotation of the matrix in Eq S.19 by $-\pi/2$).

E Time series analysis of SPDR

The temporal fluctuations of the sector indices in the SPDR represent potentially useful information about changing economic conditions. As a preliminary demonstration of the type of analysis that may be interesting in this context, we consider a retrospective analysis of how local temporal fluctuations in the market are reflected in the subspace eigenvectors of the FIM.[3]

To do this, we take a long temporal window $t = 256$ days that allows us to obtain a precise estimate of the distribution of configurations in a time window $p_{\rm win}(s)$. Then, we minimize the KL divergence between $p_{\rm win}$ and the pairwise maxent model solved on the entire data set with change in the couplings $\tilde{J}_{\rm ij} = J_{\rm ij} + \eta\Delta J_{\rm ij}$ constrained to be along the principal stock index subspace eigenvector $\Delta J_{\rm ij}$ by adjusting the coefficient η,

$$\eta^* = \arg\min_{\eta} \sum_s p_{\rm win}(s) \ln\left(\frac{p_{\rm win}(s)}{p(s;\{J_{\rm ij}+\eta\Delta J_{\rm ij}\})}\right). \tag{S.22}$$

[3]Note that the subspace eigenvectors are calculated from the entire time series available, whereas realtime analysis would rely only the statistics available up to the current time. This is why we call this example "retrospective."

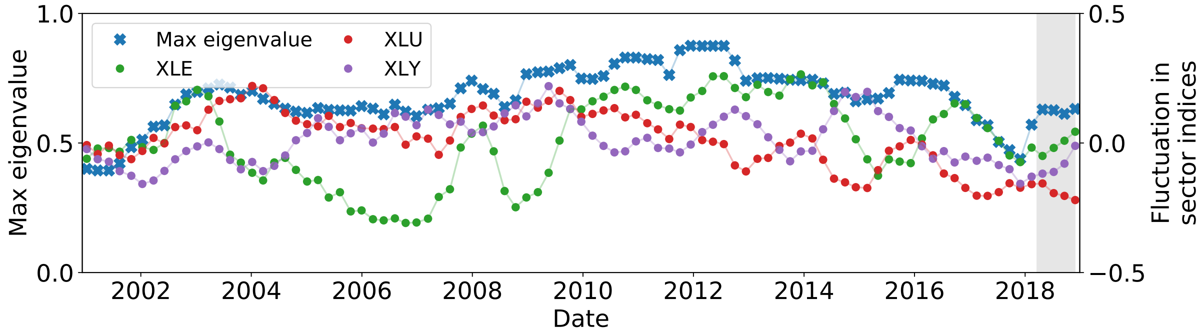

Figure S.11: Retrospective time series analysis of the SPDR for the two most pivotal indices XLE and XLU and least pivotal XLY. We use a moving windowed window of duration $t = 256$ days and a shift of $\Delta t = 50$ days. The width of the moving window is delimited by the gray box. We compare the maximum of the normalized eigenvalues of the covariance matrix with η^*, the projection of the windowed time series fluctuation onto the stock index principal subspace eigenvector (Eq S.22). Lines are drawn for readability.

The magnitude of this coefficient $|\eta^*|$ is a measure of how strongly the fluctuations in the windowed time series are reflected in the linearized direction of parameter space specified by the subspace eigenvector. As we show in Figure S.11, the fluctuations show patterns that diverge at many points from the maximum of the normalized eigenvalue of the windowed covariance matrix, a measure used to determine when economic conditions are changing [13]. In particular, we note that periods of time where the best fit value η^* between the various stock indices are correlated or anti-correlated may be useful indicators. Although it remains to relate these patterns to recognized features of the time series, this presents a potentially useful complement to existing tools for analyzing market data.[4]

F Comparison of CA state and federal legislatures

Are institutional differences captured in our measures of the eigenvectors of the FIM? As an example, we compare the CA Assembly and Senate with the US House of Representatives and Senate using our measures of the dominant pivotal measure and total asymmetry defined in Figure S.12.

When we inspect the distribution of the principal pivotal component $\tilde{\lambda}_{\max}$, we find significant distinction between the state vs. federal levels. With the Kolmogorov-Smirnov (KS) test — testing whether or not the largest difference between the two sample cumulative distribution functions (CDF) rules out a coincident underlying distribution — summarized in Figure S.13,

[4]We do not discuss in detail here the difficulty of estimating information quantities in the limit of small data, an important issue for realtime forecasting of changing economic conditions. Entropy estimation for small samples remains an active research problem [6, 14], and we avoid this issue by taking long windows.

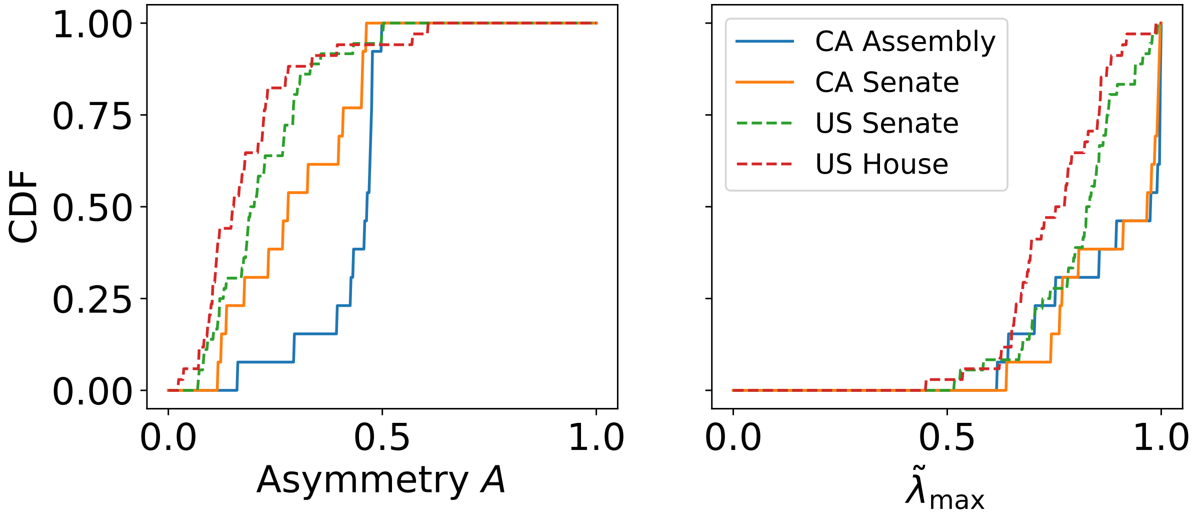

Figure S.12: Comparison of the cumulative distribution functions (CDF) of (left) the total asymmetry A and (right) the dominant pivotal measure across CA state legislatures and the US House of Representatives and Senate. We compare the distributions of $\tilde{\lambda}_{\max}$ in Figure S.13.

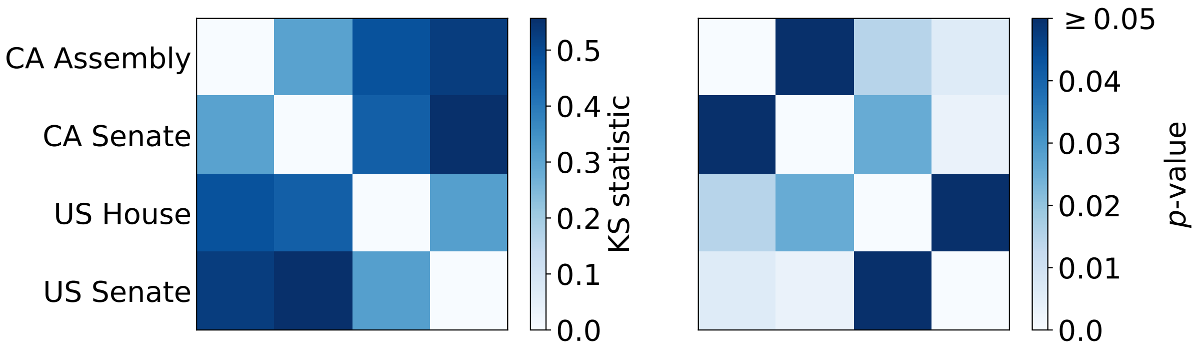

Figure S.13: Results of Kolmogorov-Smirnov test on the dominant pivotal measure across CA state legislatures and the US House of Representatives and Senate.

Bloc 1	Bloc 2	Bloc 3	Bloc 4	Bloc 5	Bloc 6	Bloc 7	Bloc 8	Bloc 9
Migden	Corbett	Knox	Cardenas	Dutra	Reyes	Robert Pacheco	Olberg	Brewer
Aroner	Cedillo	Hertzberg	Lowenthal	Mazzoni	Havice	Leach	Strickland	Ashburn
Bock	Keeley	Torlakson	Ducheny	Wright	Florez	Dickerson	Campbell	Leonard
Romero	Firebaugh	Strom-Martin	Vincent	Nakano	Cunneen	Cox	Briggs	Baugh
Kuehl	Villaraigosa	Alquist	Thomson	Wayne	Maldonado	Maddox	Runner	Ackerman
Longville	Washington	Gallegos	Davis	Papan	Pescetti	Rod Pacheco	Aanestad	Thompson
Wildman	Wiggins	Calderon	Lempert	Machado	Granlund	Battin	Oller	Baldwin
Shelley	Honda	Wesson	Scott	Cardoza	Zettel	Margett	House	Kaloogian
Steinberg		Jackson		Correa		Bates		McClintock

Figure S.14: Names of congressmen and congresswomen in CA Assembly 1999 session by voting bloc as determined by ranking on first W-Nominate dimension. Though all members were included for the W-Nominate analysis, only members who voted in more than 20% of the recorded votes were included for the coarse-graining and maxent solution.

we show the KS statistics to be larger and the p-values smaller between state and federal levels. Thus, the distribution of the principal pivotal measure is one way of distinguishing between the different levels of legislature. Notwithstanding further questions about the choice of coarse-graining — which was implemented following the same procedure outlined for the CA legislatures in Methods — our findings suggest that the structure of CA state legislatures makes them more conducive to the presence of pivotal voting blocs that precisely determine the distribution of majority-minority coalitions.

After measuring the total asymmetry for all the sessions we solved, we find that the distributions of total asymmetry for the House and the Senate are strongly similar, but those of the CA Assembly and CA Senate are not. We find that the large difference between the House and the CA Senate and between the Senate and the CA Senate is not statistically significant, given the number of data points. However, all three are significantly different from the CA Assembly with $p < 0.05$, suggesting that some systematic, institutional pattern might be gleaned from inspecting total asymmetry.

References and Notes

[1] Duncan Black. On the Rationale of Group Decision-making. *J. Political Econ.*, 56(1):23–34, 1948.

[2] Edward D. Lee. To be published. 2019.

[3] Edward D. Lee, Chase P. Broedersz, and William Bialek. Statistical Mechanics of the US Supreme Court. *J. Stat. Phys.*, 160(2):275–301, July 2015.

[4] E. T. Jaynes. Information Theory and Statistical Mechanics. *Phys. Rev.*, 106(4):620–630, May 1957.

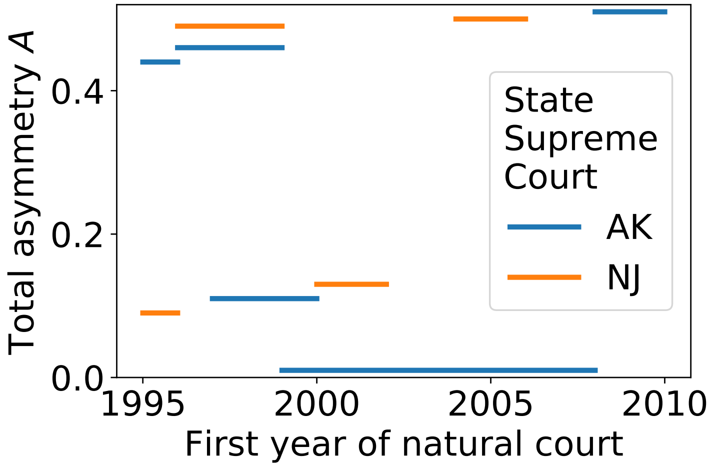

Figure S.15: Total asymmetry for the natural courts in the AK and NJ Supreme Courts by the years they were in session. We show the calculated asymmetry with lines spanning the first to last years on record for a full vote (including every sitting justice). There is overlap between natural court years because some of the data are mislabeled and show justices participating in votes after their official date of retirement, and we take the dates as given in the data set.

[5] Edward D. Lee and Bryan C. Daniels. Convenient Interface to Inverse Ising (ConIII): A Python 3 Package for Solving Ising-Type Maximum Entropy Models. *JORS*, 7(1):3, March 2019.

[6] William S. Bialek. *Biophysics: Searching for Principles*. Princeton University Press, Princeton, NJ, 2012.

[7] William Bialek and Rama Ranganathan. Rediscovering the power of pairwise interactions. *arXiv:0712.4397 [q-bio]*, December 2007.

[8] Lina Merchan and Ilya Nemenman. On the Sufficiency of Pairwise Interactions in Maximum Entropy Models of Networks. *J. Stat. Phys.*, 162(5):1294–1308, March 2016.

[9] Thomas M. Cover and Joy A. Thomas. *Elements of Information Theory*. John Wiley & Sons, Hoboken, 2nd edition, 2006.

[10] Katherine N. Quinn. *Patterns of Structural Hierarchies in Complex Systems*. PhD thesis, Cornell University, 2019.

[11] Mark K. Transtrum and Peng Qiu. Model Reduction by Manifold Boundaries. *Phys. Rev. Lett.*, 113(9):098701, August 2014.

[12] R. K. P. Zia, Edward F. Redish, and Susan R. McKay. Making Sense of the Legendre Transform. *Am. J. Phys.*, 77(7):614–622, July 2009.

[13] Michael J. Bommarito and Ahmet Duran. Spectral analysis of time-dependent market-adjusted return correlation matrix. *Physica*, 503:273–282, August 2018.

[14] Ilya Nemenman, F. Shafee, and William Bialek. Entropy and Inference, Revisited. In T. G. Dietterich, S. Becker, and Z. Ghahramani, editors, *Advances in Neural Information Processing Systems 14*, pages 471–478. MIT Press, 2002.
